# Surgical Management of a Giant Inflammatory Hepatocellular Adenoma in a Young Female

**DOI:** 10.7759/cureus.62097

**Published:** 2024-06-10

**Authors:** Atl Simon Arias Rivera, Anaida Xacur Trabulce, Moises Brener Chaoul, Marco A De La Rosa Abaroa, Rafael Padilla Longoria

**Affiliations:** 1 General Surgery, Hospital Angeles Lomas, Huixquilucan, MEX; 2 Surgical Oncology, Hospital Angeles Lomas, Huixquilucan, MEX

**Keywords:** young female, resection, right hepatic lobule, liver, hepatocellular adenoma

## Abstract

Hepatocellular adenomas are rare and benign primary neoplasms of phenotypically mature hepatocytes. Our understanding of this pathology has greatly improved due to advances in molecular and anatomic knowledge. This article provides an in-depth review of hepatic adenomas (HCA) while presenting the case of a 20-year-old patient with a giant inflammatory hepatocellular adenoma with an atypical presentation, in whom surgical intervention was performed via right hepatectomy. In the post-surgical course, the patient had an in-hospital stay of three days with no complications. During outpatient monitoring via laboratory tests and imaging at eight months, the patient did not present any trace of recurrence.

## Introduction

Hepatic adenomas (HCA), also known as hepatocellular adenomas, are rare and benign primary neoplasms. Hepatic adenomatosis is defined as the presence of more than 10 adenomas, with multiple lesions reported in 12% to 30% of cases. In the 1970s, after the introduction of oral contraceptives, the prevalence of liver cell adenomas (LCAs) increased since the pathogenesis is associated with steroid and anabolic hormone use. HCA can predominantly be found in young women aged between 20 and 40 years, with a female to male ratio of 11:1. They have an incidence rate of approximately 3 cases per 100,000 individuals [1-3].

The prevalence of symptoms in 50-75% of patients makes upper abdominal pain the most often observed symptom, usually associated with hemorrhage or local compression. Complications of this condition include bleeding, which occurs in 15-20% of cases, and malignant transformation, which occurs in 5% of cases. The 5-7 cm rise in tumor size necessitates the suggestion to surgically remove all HCAs that are greater than 5 cm [3-4].

There are five main subtypes of hepatocellular adenoma: HNF1a-inactivated HCA (HHCA), Inflammatory HCA (IHCA), β-catenin-activated HCA (BHCA), Sonic Hedgehog HCA (shHCA), and less than 5% of HCA will be unclassified because of the absence of molecular abnormalities or immunohistochemistry markers [4].

Major complications of hepatocellular adenoma include rupture, with an estimated risk of 30% to 50%, which mostly occurs in lesions 5 cm or larger. Hemorrhage can be managed with hepatic artery embolization. Malignant transformation is also possible; in the molecular-pathologic classification, the subtype with the highest risk of malignancy is the LCA with β-catenin activation. It is established that CTNNB1 oncologic mutations have the capacity to activate β-catenin at high, moderate, or weak levels, each predisposing to different tumor phenotypes and malignant progression [3-6].

We present a case of a 20-year-old patient with a giant inflammatory hepatocellular adenoma with an atypical presentation.

## Case presentation

A 20-year-old female was referred to the surgical oncology department due to a hepatic mass. During the anamnesis, she denied any past utilization of oral contraceptives, alcohol intake, consumption of bee syrup, or history of hepatitis. Previously asymptomatic, after facing difficulties losing weight, she was taken by her mother to perform a routine blood test examination. Pancreatic function tests revealed a significant elevation in amylase (260 U/L) and lipase (566.2 U/L), with liver function tests within normal range.

**Figure 1:**
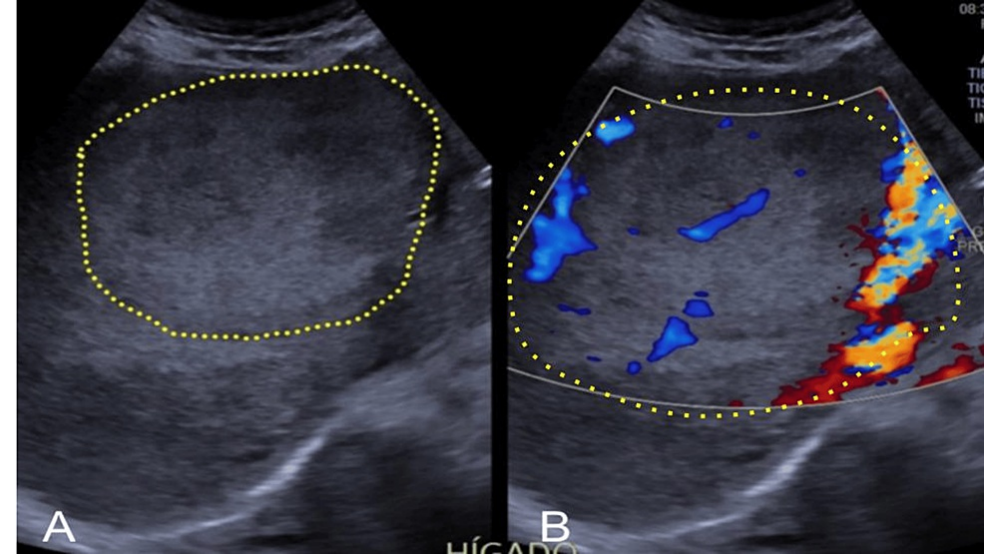
A-B: Solid, heterogeneous, irregular, and giant neoplastic lesion (yellow line) measuring 150 x 137 x 26 mm, almost totally replacing the right hepatic lobe, with increased peripheral vascular activity.

During the clinical examination, a large palpable abdominal mass was discovered, approximately 15 cm in diameter. Subsequent abdominal ultrasonography revealed a solid, heterogeneous, irregular neoplastic lesion in the liver measuring 150 x 137 x 26 mm. This lesion nearly entirely occupied the right hepatic lobe and showed increased peripheral vascular activity (Figure 1), a finding confirmed by magnetic resonance imaging. Serum biomarkers alpha-fetoprotein, CA 19-9, and carcinoembryonic antigen were within normal range (Table 1). The serum C-reactive protein (CRP) was 6.4 mg/dL (Table 2). There were also elevated values of amylase, lipase, alkaline phosphatase, and gamma-glutamyl transferase (Table 2).

**Table 1:** Preoperative tumor cell markers. Results within normal parameters.

Tumor Marker	Result	Reference Range
Alpha-fetoprotein	1.7 ng/mL	<8.8
Carcinoembryonic antigen	1.79 ng/mL	0.00-5.00
CA 19-9	17.7 U/mL	0.0-37.0

**Table 2:** Preoperative laboratory results.

Laboratory test	Result	Reference Range
Hemoglobin	11.96 g/dL	12.00-16.00
Hematocrit	33.9%	37.0-48.0
WBC Count	11,930 10^3^/μL	3.80-11.20
Lymphocytes	18%	20-40
Neutrophils	65%	40-70
Stab neutrophils	10%	0.0-4.0
Platelets	262,000 10^3^/μL	130-450
Glucose	82.3 mg/dL	74.0-99.0
Urea	9.5 mg/dL	10.7-53.5
Creatinine	0.75 mg/dL	0.60-1.60
Uric acid	2.9 mg/dL	2.6-6.0
Lactate dehydrogenase	506.1 U/L	125.0-243.0
C-reactive protein	6.42 mg/dL	0.010-0.744
Creatine phosphokinase	4540.9 U/L	29.0-168.0
Alanine Aminotransferase	317.7 U/L	0.0-55.0
Aspartate Aminotransferase	407.3 U/L	5.0-34.0
Alkaline phosphatase	237.0 U/L	40.0-150.0
Gamma-glutamyl transferase	81.1 U/L	9.0-36.0
Total bilirubin	1.09 mg/dL	0.20-1.20
Direct bilirubin	0.50 mg/dL	0.0-0.50
Indirect bilirubin	0.59 mg/dL	0.0-1.00
Amylase	1032.5 U/L	25.0-125.0
Lipase	35.7 U/L	8.0-78.0
International Normalised Ratio	1.17 sec	0.80-1.20
Prothrombin time	14.1 sec	10.0-13.9
Partial Thromboplastin time	45 sec	24.0-40.0

**Figure 2:**
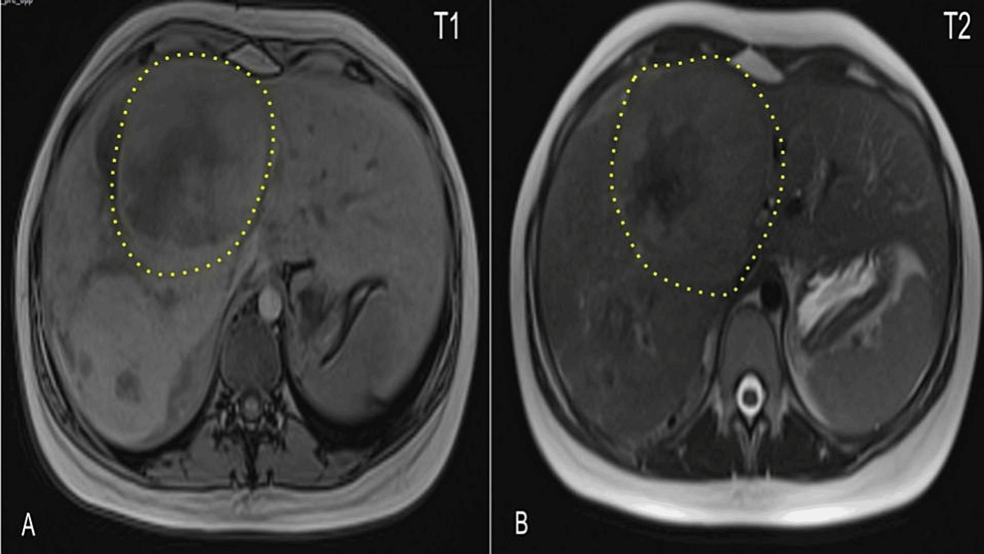
A) In the magnetic susceptibility sequence, irregular hypointense areas with a central distribution suggestive of blood remains are identified. In the diffusion sequence, no restriction areas are identified. B) After the application of intravenous gadolinium, the lesion shows heterogeneous avid enhancement, predominantly peripheral in the arterial phase, with moderate washout in the venous and equilibrium phases. Hepatic tumor is indicated by yellow-dotted line.

**Figure 3:**
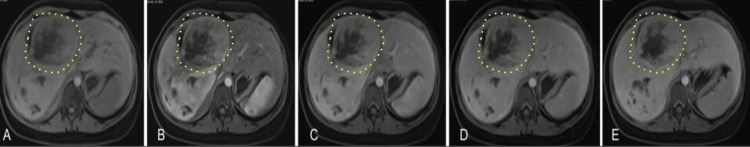
Dynamic sequence with hepatospecific gadolinium: A) Pre-contrast 0''; B) Arterial phase 25-30''; C) Venous phase 40-45''; D) Balanced phase 20''; and E) Late phase 30''. Hepatic tumor is indicated by yellow dotted line.

MRI with T1 and T2 sequences reported a liver with a longitudinal axis of 19.9 cm. The right hepatic lobe had an oval, encapsulated, large lesion with dimensions of 13.7 x 14.6 x 19.4 cm and an estimated volume of 2020 cc. The borders of the tumor were well-defined, occupying segments V, VII, and VIII, with a heterogeneous pattern at the expense of cystic and necrotic areas (Figure 2). After IV Gadolinium administration, there was a heterogeneous avid enhancement with peripheral predominance in the arterial phase and moderate lavage in the venous and equilibrium phases (Figure 3). The lesion partially compressed the inferior vena cava, displaced the pancreas to the contralateral side, and pushed the right kidney caudally. It was not associated with free fluid or reactive lymph nodes.

**Figure 4:**
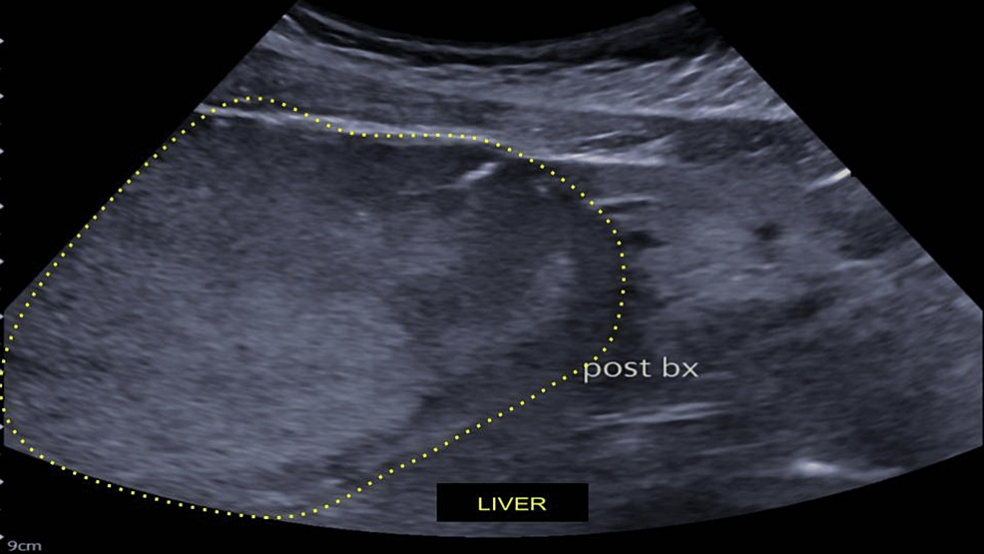
Liver appearance post-ultrasound-guided biopsy. No hematoma detected after biopsy.

The USG-guided liver biopsy reported a hepatic lesion compatible with hepatocellular adenoma of the inflammatory subtype (telangiectatic). The immunophenotype was glutamine synthetase positive and amyloid A positive, with a proliferation rate of <1% (Figure 4).

**Figure 5:**
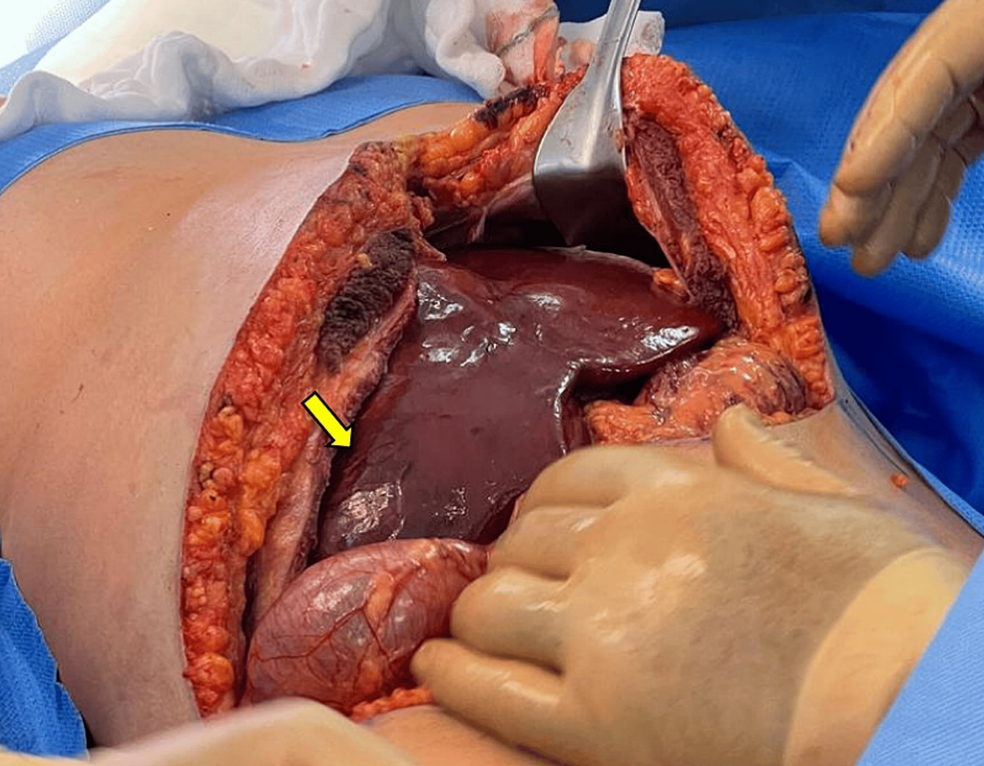
Chevron incision in the abdominal wall with adequate exposure of the liver and evidence of a tumor in the right hepatic lobule (indicated by yellow arrow).

**Figure 6:**
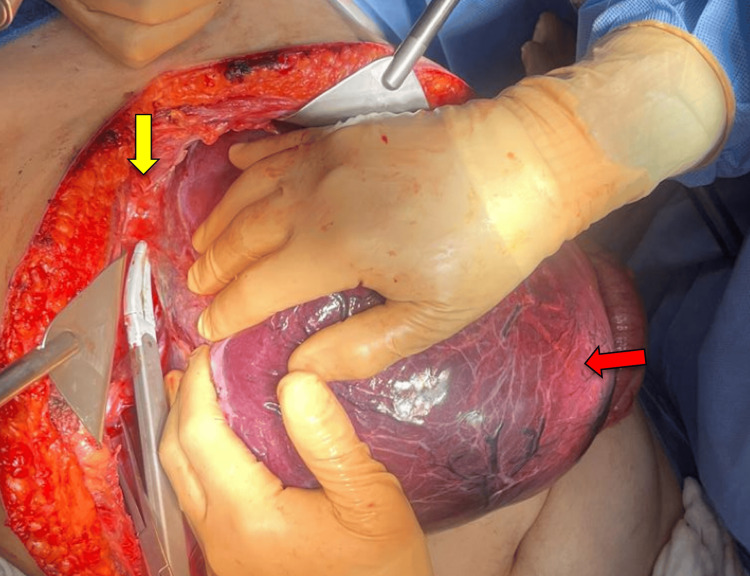
Releasing the right coronary and triangular ligaments (yellow arrow) using bipolar energy, with the tumor being retracted (red arrow).

**Figure 7:**
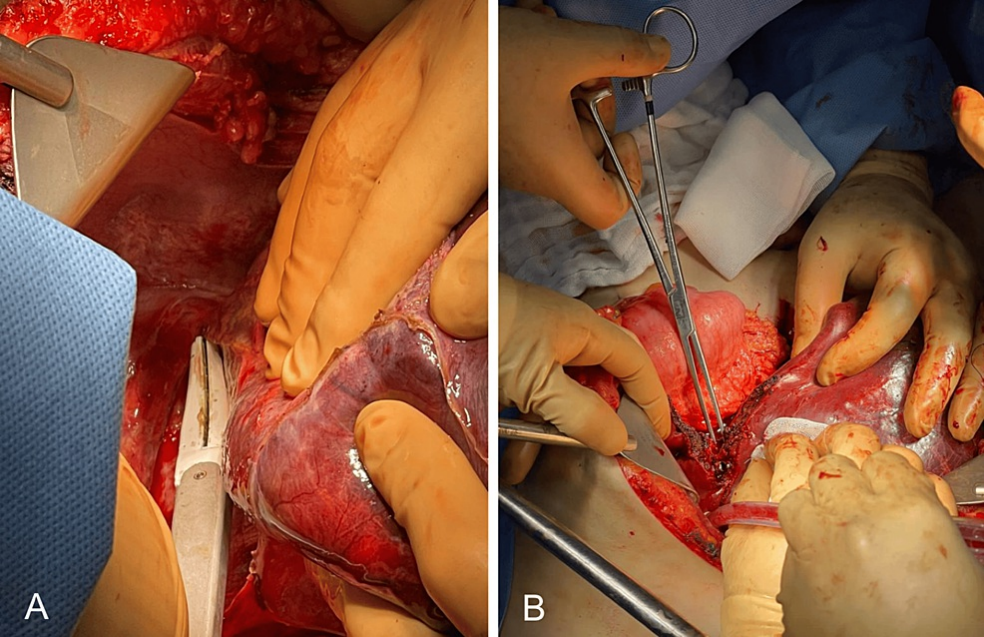
A-B: Separation of the right hepatic lobe using bipolar energy, facilitated by an impact and extraction clamp. Dissection of liver parenchyma with bipolar energy.

**Figure 8:**
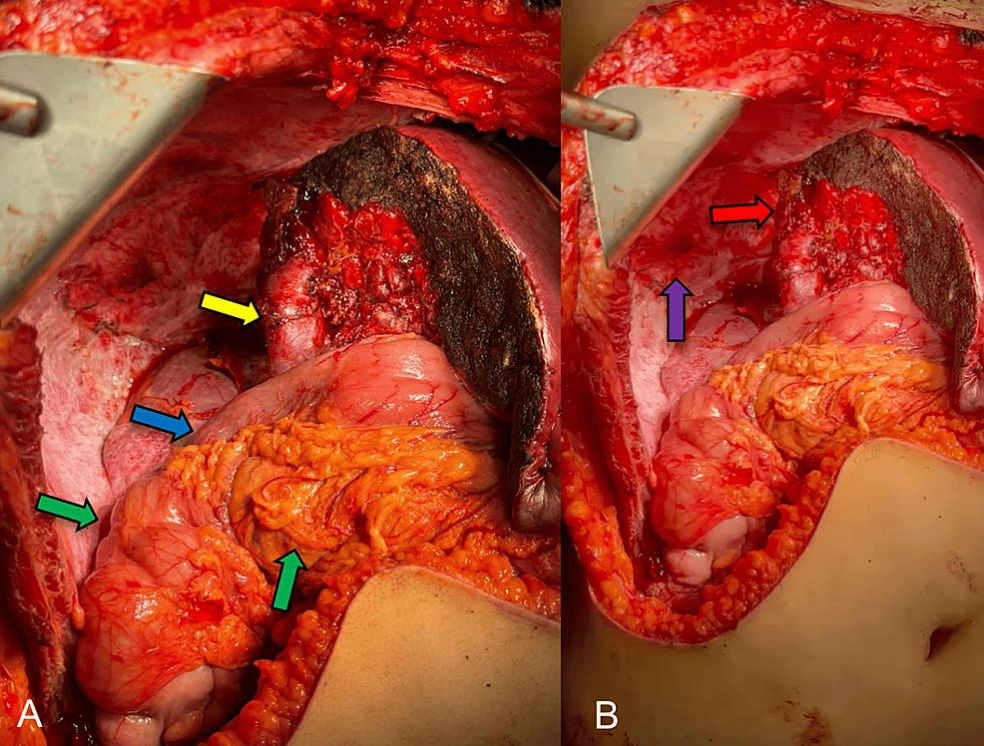
Posterior-to-right hepatectomy with adequate hemostasis of the remaining liver. Identifications include the portal vein (yellow arrow), middle suprahepatic vein (red arrow), colon (green arrow), duodenum (blue arrow), and diaphragm (purple arrow).

**Figure 9:**
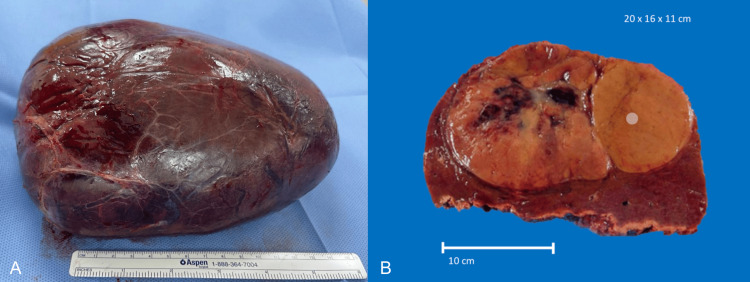
Macroscopic appearance of the resected specimen showing a non-encapsulated, well-defined, solitary tumor with central hemorrhage on the cut surface. Dimensions: 20 x 26 x 11 cm.

A surgical intervention was performed via right anatomic hepatectomy using a chevron incision through the abdominal wall (Figure 5). An automatic abdominal retractor was placed for better exposure. The release of the right coronary and triangular ligaments was achieved with bipolar energy (Figure 6). Resection of the right hepatic lobe was completed with monopolar and bipolar energy, aided by intraoperative ultrasound (Figures 7-8). The Pringle maneuver was not necessary during the surgery. The surgery did not have any complications. The right hepatic lobe measured 20 x 16 x 11 cm and was indurated upon palpation, with a heterogeneous superficial pattern and a more vascularized zone (Figure 9). The abdominal drain was placed in the right subdiaphragmatic space. In the post-surgical period, the patient had an in-hospital stay of 3 days without any complications.

During the outpatient monitoring via laboratory tests and imaging at the first and third months, the patient did not exhibit any disease recurrence. A total of six months of follow-up was conducted with no post-surgical complications.

**Figure 10:**
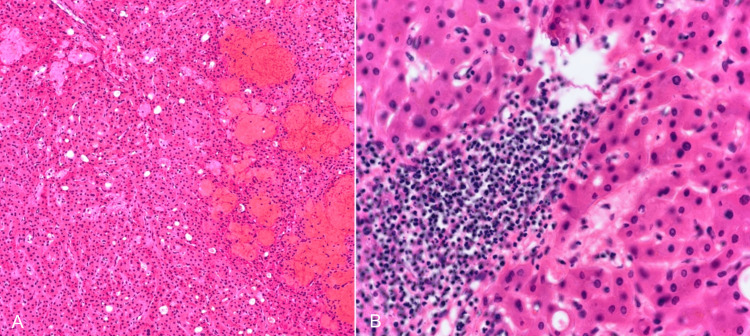
A-B: The slides reveal a tumor displaying a clearly delineated border with the surrounding healthy liver tissue. It consists predominantly of hepatocytes exhibiting no notable cytologic abnormalities. Several areas exhibit hemorrhage and necrosis. There is no presence of cytologic atypia, unusual mitosis, or invasion into parenchymal regions. Ductular reaction is present.Several areas display hemorrhage and necrosis. There are no signs of cytologic atypia, unusual mitoses, or invasion into parenchymal regions. A ductular reaction is present.

Pathology examination confirmed the diagnosis of hepatocellular adenoma, telangiectatic inflammatory subtype. The presence of areas exhibiting hemorrhage and necrosis without atypia, unusual mitosis, or invasion into parenchymal regions was noted (Figure 10).

## Discussion

HCAs are infrequent, noncancerous tumors originating from hepatocytes, manifesting at a rate of approximately 3-4 occurrences per 100,000 individuals [7].

These tumors typically range in size from 8 to 15 centimeters, although reports of giant adenomas exceeding 20 cm are rare [8]. Their growth is closely linked to hormonal levels. Approximately half of hepatic adenomas exhibit regression or cessation of growth upon discontinuation of oral hormonal therapy [8]. The patient denied any history of oral hormonal medication usage.

The primary complications associated with HCAs include potential malignant progression, hemorrhage, and rupture, with their occurrence becoming more likely as the adenoma size increases [9-10]. When dealing with giant adenomas, the optimal treatment modality is surgical removal, performed at a liver surgery center with expertise in such procedures [8].

The classification of HCAs into subtypes relies on histopathological assessment, which considers the unique molecular features of each subtype. Accurate diagnosis is vital because clinical manifestations, such as the likelihood of malignancy, vary according to the subclassification [11].

IHCA is linked to conditions such as excessive alcohol consumption, metabolic syndrome, and obesity, along with heightened levels of inflammation-related proteins like serum amyloid A (SAA) and CRP [12-13]. Despite being overweight, our patient did not meet the criteria for obesity and denied any significant alcohol consumption during questioning.

The distinction between various liver tumors is crucial to clinical practice due to differences in medical management approaches. For instance, focal nodular hyperplasia (FNH) shares similarities with HCA, yet surgical intervention is typically unnecessary for FNH unless the patient experiences persistent pain that does not respond to treatment [11].

For diagnosing HCA through radiological means, multiphase dynamic contrast-enhanced MRI or multiphase CT are commonly employed, with the former being the preferred modality [14-15]. HCAs typically appear hypoattenuating on noncontrast CT scans, exhibit hypervascularity and heterogeneity during the arterial phase, and demonstrate iso/hypoattenuation during the portal venous phase of contrast-enhanced CT scans [15]. Arterial enhancement is a shared feature between HCAs and FNH, and differentiation between the two can be achieved using hepatocyte-specific contrast agents when standard imaging yields inconclusive results [16].

Laumonier H et al. and Ronot M et al. have highlighted MRI’s utility in distinguishing between the two main subtypes of HCA: HNF-1a-inactivated and inflammatory HCA, each displaying distinct features associated with intratumoral steatosis and sinusoidal dilatation, respectively [17-18].

The expected findings on multiphase dynamic contrast-enhanced MRI for inflammatory HCA are as follows in T1/T2-weighted images: presenting with isointensity or slight hyperintensity, and maintaining signal integrity on chemical shift sequences, with diffuse hyperintensity. Notable arterial enhancement continues into the portal venous and delayed phases [16].

In our case, the MRI showed, as previously mentioned, a heterogeneous avid enhancement of peripheral predominance in the arterial phase, with moderate washout in the venous and balance phases being compatible with the frequent findings in inflammatory HCA.

Hemorrhage occurs in 21% to 40% of HCAs, and in most cases, the bleeding is intratumoral. However, it can lead to tumor rupture, resulting in either intraperitoneal or subcapsular hemorrhage. The incidence of hemorrhage is higher in HCAs larger than 5 centimeters, with other contributing factors including the inflammatory subtype, tumor growth, recent hormone usage within the past six months, and pregnancy [19]. Emergency surgery for ruptured HCAs carries a mortality rate of 5% to 10% [20].

The probability of HCA transitioning into malignancy is approximately 5%, influenced by factors such as patient gender, tumor size, and subtype [21]. Mutations in the ß-catenin gene are correlated with an increased likelihood of malignant transformation, with up to 46% of ß-catenin-activated HCAs showing characteristics indicative of hepatocellular carcinoma (HCC) [22]. About 10% of inflammatory HCAs with mutated ß-catenin genes may undergo malignant conversion [23].

For male patients, surgical resection is advised irrespective of tumor size, while for female patients, resection is recommended for tumors measuring 5 cm or larger. However, for multiple tumors smaller than 5 centimeters, surgical resection is not deemed necessary, as most residual HCAs tend to remain stable or even regress spontaneously [21].

Transarterial embolization (TAE) is a recommended intervention for cases of HCA complicated by hemorrhage. Patients experiencing tumoral hemorrhage rarely exhibit hemodynamic instability, allowing for an initial TAE procedure before delayed surgery. TAE is indicated within 2 to 3 days following tumoral hemorrhage, preferably using a selective approach [21-24]. This procedure is minimally invasive, can be safely repeated, and has shown promising outcomes; approximately 80% of tumors may exhibit stability, regression, or complete involution. Importantly, these favorable results are primarily observed in cases involving small tumors. Additionally, TAE may be considered for managing large, multiple, or bilateral tumors, as well as for preoperative reduction in tumor size [24].

The patient presented with a large tumor measuring over 20 cm in diameter, which posed a higher risk of hepatic rupture rather than malignant change. The decision was made to proceed with surgical management, and TAE was not employed.

## Conclusions

In conclusion, HCAs are tumors for which the size and subtype dictate the surgical management. Likewise, the treatment of choice for giant HCAs is surgery, which most of the time results in practically no recurrence after surgical resection. Also, the prompt diagnosis of this tumor can diminish the complications that the patient may experience in post-surgical recovery.
